# Determinants for refusal of HIV testing among women attending for antenatal care in Gambella Region, Ethiopia

**DOI:** 10.1186/1742-4755-9-8

**Published:** 2012-07-26

**Authors:** Wondimagegn Fanta, Alemayehu Worku

**Affiliations:** 1 United Nations Population Fund (UNFPA), Addis Ababa, Ethiopia; 2 School of public health, Addis Ababa University, Addis Ababa, Ethiopia

**Keywords:** HIV testing, Women attending antenatal care service, Counseling and testing

## Abstract

**Background:**

In Gambella region, inhabitants owe socio-cultural factors that might favor refusal for HIV testing service utilization among Antenatal Care attendees.

**Objective:**

To assess determinants for refusal of HIV testing service utilization among ANC attendees in Gambella Region.

**Methods:**

A comparative cross sectional study was conducted among ANC attendees from March 2008 to May 2008 in four selected health facilities of Gambella region. Sample size of 332 participants (83 who refused HIV testing and 249 who accepted HIV testing) were taken for the study. The study was supplemented with four focus group discussions. Multivariate binary logistic regression was employed to control for confounding factors.

**Results:**

When adjusted with other factors pregnant women with 2–3 live births in the past; who claimed divorce as a perceived response of their husband following HIV positive test result; who had not sought agreement from their husband for testing; disclosure of test for husband and being from certain ethnic group (E.g. Mejenger) were independent predictors for refusal of HIV testing among ANC attendees.

**Conclusion and recommendation:**

Based on the findings, the following recommendations were forwarded: Provision of innovative information and education on the pre-test session for those pregnant women having two or more children; community involvement to tackle stigma; women empowerment; designing couple friendly counseling service; and fighting harmful traditional practices related with decision of HIV testing.

## Introduction

In Gambella region, where the study was conducted, the total adult prevalence rate of HIV infection in 2007 was 2.4% with estimated HIV positive pregnant mothers of 251 and annual HIV positive births of 47 [1]. The estimated number of persons living with HIV worldwide in 2007 was 33.2 million. Sub-Saharan Africa continued to be the region most affected by the AIDS pandemic. There were an estimated 22.5 million people living with HIV in the region in 2007.. The national single HIV prevalence rate was estimated to be 2.1% for the year 2007 [1,2]. According to ANC surveillance results, HIV prevalence among pregnant women aged 15–24 in Ethiopia declined from 5.6% in 2005, to 3.5% in 2007, and then to 2.6% in 2009. The Ethiopia Demographic and Health Survey 2011 data show an overall prevalence of 1.5% among the general population. The HIV estimates of Ethiopia show 789,900 people currently living with HIV/AIDS (607,700 adults and 182,200 children aged 0–14 years); and 952,700 AIDS orphans [3,4].

According to a study done in two ANC clinics in Addis Ababa, the overall refusal rate for HIV screening was 27.5%. Of those refusing testing, 40% said they were afraid to be tested for HIV, 19% had already been tested, 13% felt they were healthy so the test was unnecessary, 11% did not want the test, 9% were told to refuse by family, and 8% refused for other reasons including spiritual beliefs and medical problems [5]. In another study done in Eastern part of Ethiopia, marital status; knowledge about HIV, mother to child transmission and VCT; attitude towards VCT; antenatal care follow up and perceived benefits of VCT were independent predictors of acceptance of voluntary HIV testing [6]. In Gambella region, the share of those who receive HIV testing during their ANC visit to the health facilities is 6.9%. Of all that are provided testing service, those who came back to receive their test results accounted only 0.7% [7].

Mother-to-child transmission could occur during pregnancy, labor and delivery; and after childbirth by breastfeeding. The prevention of HIV transmission from mothers living with HIV to their infants is built around the routine offer of HIV counseling and testing to all pregnant women along with other interventions [8]. Provider-initiated routine counseling and testing using the opt-out approach is recommended for all clients seen within the context of maternal care in Ethiopia [9]. This will be implemented with the routine ANC services. The level of ANC attendance in Gambella is reported to be 36.6% [10]. In PMTCT program, World Health Organization promotes a comprehensive strategic approach for the prevention of HIV infection in infants and young children. These are primary prevention of HIV infection; prevention of 3 unintended pregnancies among women living with HIV; prevention of HIV transmission from mothers living with HIV to their infants; and treatment, care, and support of HIV infected women, their infants and their families. The prevention of HIV transmission from mothers living with HIV to their infants is built around the routine offer of HIV testing and counseling to all pregnant women; counseling on infant feeding supporting exclusive breastfeeding; safer obstetrical practices; and ensuring availability of antiretroviral drugs and other supplies for PMTCT [8].

Various factors have been sited out to be associated with refusal of HIV testing service utilization among ANC attendees in different areas. Even though the extent of studies that had been conducted in the area of PMTCT is minimal in the country as a whole, there are identified factors for refusal of HIV testing up take in different study areas [5]. However, the role of these identified factors as determinants for refusal of service utilization in the context of Gambella region had not been studied previously. Besides, as Gambella region inherently owes inhabitants having different socio-cultural factors from the previous study areas, it is possible for ANC attendees to have concerns peculiar to themselves.

This study used health belief model as a conceptual framework to assess determinants for refusal of HIV testing service utilization among ANC attendees in Gambella region. Once determinants had been identified from this study, they would serve as an input for effective PMTCT program implementation in the region as well as in areas with similar set up [11].

## Methods

The study was conducted in Gambella region that has largely a hot climatic zone. Administratively, the region is divided into three zones and eleven districts. It harbors different ethnic groups. The majority of the ethnic groups residing in the region include Nuier, Agnuhak, and Mejenger. However, there are also other ethnic groups including settlers from other parts of the country (Kembata, Amhara, and Tigre) and refugees from Southern Sudan. The region has one hospital, eight health centers, thirty-five health station, twenty-two health posts, and seven private clinics. The PMTCT program was launched in 2004 at Gambella Hospital. Presently the service is being provided in one hospital, two health centers (Itang H/C and MettiH/C), one faith-based health center (Abobo H/C), and in two heath centers located at refugee camps [7].

Both quantitative and qualitative study was used in this study. For the quantitative study, a comparative cross sectional study design was conducted among ANC attendees from March to May 2008. The sample size was determined by using the formula for the difference between two population proportions. Knowledge of breastfeeding as means of MTCT of HIV was used as the variable of exposure to calculate the sample size. An assumption of estimated proportion of women who knew breastfeeding as means of MTCT of HIV to be 26% among those who were not HIV tested on their current ANC visit, and 44.8% among those who were HIV tested was taken [12] . A 5% type I error, and 80% power to detect the difference on exposure between HIV tested and not tested were assumed. Three HIV tested for each not HIV tested were taken to increase the power of the study. Accordingly, a sample size of 332 study participants of which 83 (those who were not HIV tested during the current ANC visit) and 249 (those who were HIV tested during the current visit in study period) was taken for the study.

All public health facilities except refuge health centers, where ANC and HIV counseling and testing services, wereintegrated as component of PMTCT program and located in the woredas where the dominant ethnic groups of the region reside, were included in the study. Accordingly, Gambella hospital, Metti H/C (305kms away from Gambella town), Itang H/C (53kms), and Abobo H/C (45kms) were included. The sample population from each health facility was taken by distributing the total sample size in to the respective health facilities proportionate to the size of pregnant mothers who utilized the service before the study, based on PMTCT report obtained from regional health bureau. Study subjects were pregnant women attending mother and child health care (MCH) unit, for the first antenatal care follow up who had pre-test counseling irrespective of their testing. Subjects were identified as HIV test refusal and acceptor based on the information obtained from the client card. Women came for the first ANC service of the current pregnancy who had no previous HIV testing were included in the study. The clients’ self-report whether they were tested or not in the past was also used to include or exclude clients in the study at times when their cards were missed.. Then pregnant women who attended ANC during data collection time were taken consecutively based on the size that was set for each health facility. The procedure continued throughout the data collection period until the required sample size was obtained.

Data was collected using a questioner that was translated from English to Amharic and back to English to check for its consistency. Informed consent was obtained from all eligible participants after explaining the objectives of the study. A local language speaking trained research assistant fluent in Amharic verbally administered the pre tested standard questioner to each participant in a private room on their exit. Eight interviewers and four supervisors were employed for data collection. The interviewers were nurses who work in another health institutions and the supervisors were Head of MCH unit in the respective data collection sites.

For the qualitative study, Focus Group Discussions (FGDs) were conducted in all health facilities among both groups of women until the point of redundancy. The discussants were 6–8 ANC attendees in each site. FGD guidelines were used as a tool. Information from the qualitative study were reserved to further explain determinants obtained from the quantitative findings. Moreover, the findings of the qualitative study were used to identify the felt needs of pregnant mothers on ways to improve service utilization during their ANC.

Supervision was made during the data collection process. Filled questioners were checked thoroughly for completeness and consistency. Data entry and cleaning was done using Epi/Info version 3.3. The data was exported to SPSS version 15.0 where recoding, transforming, and re-categorizing of variables were done. Frequencies of exposure variables were determined by cross-tabulation with the outcome variable. Odds ratio with 95% confidence interval and P- value were calculated to reveal statistical significance. Further analysis was done to check association between the variables of the Health Belief Model, the theoretical variables against refusal of HIV testing. To do this, bivariate analysis was first employed to check the effect of each of the predictors on the outcome of interest. Those variables with P < 0.2in the bivarite were included to the multivariate binary logistic regression model. Multivariate binary logistic regression was employed to control the effect of confounding factors. Qualitative data analysis started by transcribing and then followed by translating Amharic to English. The summary part of the translated content was coded manually and then it was analyzed by thematic analysis.

Ethical clearance was obtained from Institutional Review Board of Addis Ababa University, Medical faculty. Permission letter to conduct the research was obtained from Gambella Regional Health Bureau. Consent was obtained from each study subject. Confidentiality and privacy was maintained.

## Results

### Socio-demographic characteristics

In this study, 337 pregnant women attending antenatal care were approached and three hundred thirty two (332) pregnant women were willing to be included in the study, giving a response rate of 98.5%. The respondents were from Gambella Hospital (48.2%), Itang H/center (24.1%), Metti H/center (18.1%), and Abobo H/center (9.6%). The median age of women was 23 and 22 years among those who accepted HIV testing and refused for HIV testing, respectively.. With regard to educational status, 55.4% of accepters and 48.2% of the refusals completed primary level of education (1–8 grades). Majority of the study subjects were married. Of the married, about 41.5% of husbands for accepters and 43.0% for refusals leave home frequently for more than one month. Nearly equal proportion of both groups of women were housewives. Agnuak ethnic group comprises the largest proportion of the study subjects (22.9% vs. 37.8%), followed by Nuer (24.1% vs. 18.1%) and Amhara (16.9% vs. 15.7%) for accepters of HIV testing and refusal of HIV testing among ANC attendees respectively (Table 1).

**Table 1:** Socio - demographic characteristics of ANC attendees, Gambella Region, 2008

**Variables**	**Not-HIV tested (n = 83)**		**HIV tested (n = 249)**	
	**Frequency**	**%**	**Frequency**	**%**
**Age**				
15 - 19 yrs	16	19.3	61	24.3
20 - 34 yrs	62	74.7	179	71.9
35 - 49 yrs	5	6	9	3.6
**Religion**				
Protestant	46	55.4	161	64.7
Orthodox	23	27.7	48	19.3
Catholic	5	6.0	12	4.8
Muslim	8	9.6	26	10.4
Others	1	1.2	2	0.8
**Ethnic group**				
Nuer	20	24.1	45	18.1
Agnuak	19	22.9	94	37.8
Mejenger	3	3.6	9	3.6
Oromo	12	14.5	22	8.8
Amhara	14	16.9	39	15.7
Others	15	18.0	40	16.0
**Educational level**				
Illitrate	15	18.1	72	28.9
Read and write	11	13.3	15	6
1-8 grade	46	55.4	120	48.2
9 and above	11	13.2	32	16.8
**Marital status**				
Married	78	94.0	207	83.1
Live together	4	4.8	37	14.9
Unmarried	0	0	2	0.8
Separated	1	1.2	3	1.2
**Husband staying away from**				
**home > 1 month**				
Yes	34	41.5	105	43.0
No	45	54.9	133	54.5
Refused to respond	3	3.7	6	2.5
**Income compared with the same**				
**community members**				
High	4	4.8	11	4.4
Moderate	46	55.4	154	61.8
Low	33	39.8	84	33.7

### Factors associated with HIV testing among ANC attendees: socio-demographic

Among the socio-demographic variables, lower educational level was found to have a positive association with refusal of HIV testing. The odds of refusal of HIV testing among those who read and write was nine and half times more than those who are grade 12 and above. Ethnicity also had significant association with refusal of HIV testing. A pregnant woman from the Nuer ethnic group has two-fold likelihood of refusing HIV test than a woman from the Agnuak ethnic group (COR = 2.2, 95% CI = 1.07, 4.52) (Table 2). Nevertheless, when adjusted with other factors the association turned out insignificant (AOR = 1.6, 95% CI = 0.55, 4.68). On the other hand, the adjusted odds ratio indicated that a pregnant woman from Mejenger (AOR =20.3, 95% CI =1.83, 226.01), Kembata (AOR =19.6, 95% CI =3.08, 124.97), and Oromo (AOR = 8.1; 95% CI = 2.10, 31.42) are more likely to refuse HIV testing than a woman from Agnuak ethnic group (Table 2).

**Table 2:** Socio - Demographic factors associated with refusal of HIV testing during ANC, Gambella Region, 2008

**Variables**	**Not-HIV tested**	**HIV tested**	**COR (95% CI)**	**P-Value**
**Age**				
34 - 45 yrs	5	9	2.1(0.62-7.2)	0.23
20 - 34 yrs	62	179	1.3(0.71-2.46)	0.38
15 - 19 yrs	16	61	1.0	
**Religion**				
Muslim	8	26	1.1(0.46-2.54)	0.87
Orthodox	23	48	1.7(0.93-3.04)	0.09
Catholic	5	12	1.5(0.49-4.35)	0.50
Protestant	46	161	1.0	
**Ethnic group**				
Kembata	6	9	*3.3(1.05-10.36)	*0.04
Nuer	20	45	*2.2(1.07-4.52)	*0.03
Mejenger	3	9	1.6(0.41-6.66)	0.48
Oromo	12	22	*2.7(1.14-6.37)	*0.02
Amhara	14	39	1.8(0.8-3.89)	0.15
Tigre	3	6	2.5(0.57-10.77)	0.23
Agnuak	19	94	1.0	
**Educational level**				
Illitrate	15	72	0.61(0.25-1.51)	0.38
Read&write	11	15	*2.12(0.74-6.11)	0.24
1-8 grade	46	120	1.11(0.52-2.48)	0.78
9 and above-	110	32	1.0	
**Marital status**				
Separated	1	3	0.88(0.09-8.63)	0.91
Live together	4	37	*0.28(0.1-0.83)	*0.02
Unmarried	0	2	0.01(0.00-1.3)	0.74
Married	78	207	1.0	
**Husband stay away from**				
**home > 1 month**				
Yes	34	105	0.96(0.57-1.6)	0.87
No	45	133	1.0	
**Income compared with the same community members**				
Low	33	84	1.08(0.32-3.6)	0.9
Moderate	46	154	0.82(0.25-2.7)	0.75
High	4	11	1.0	

### Factors associated with HIV testing among ANC attendees: knowledge on PMTCT

With respect to knowledge-related factors, those who did not know breastfeeding as a route of mother to child transmission were almost two times more likely to refuse HIV testing than their counter parts (COR = 2.33, 95% CI = 1.41, 3.87). However, when adjusted with some socio-demographic and practice related factors, the adjusted odds ratio indicated no association with refusal of HIV testing (AOR = 1.48, 95% CI = 0.53, 4.18) (Table 3). Similarly, respondents who did not see prevention of mother to child transmission of HIV as benefit of testing during ANC were 1.88 times more likely to refuse HIV testing than their counter parts (COR = 1.88, 95% CI = 1.14, 3.11) (Table 4).

**Table 3:** Independent predictors of refusal of HIV testing among ANC attendees, Gambella Region, 2008

**Variables**	**Not-HIV tested**	**HIV tested**	**COR (95% CI)**	**AOR (95%CI)**
**Age**				
34-45	5	9	2.1(0.62-7.2)	1.4(0.11-18.5)
20-34	62	179	1.3(0.71-2.46)	1.3(0.42-4.13)
15-19	16	61	1.0	1.0
**Ethnic group**				
Kembata	6	9	*3.3(1.05-10.36)	*19.6(3.08-124.97)
Nuer	20	45	*2.2(1.07-4.52)	1.6(0.55-4.68)
Mejenger	3	9	1.6(0.41-6.66)	*20.3(1.83-226.01)
Oromo	12	22	*2.7(1.14-6.37)	*8.1(2.1-31.42)
Amhara	14	39	1.8(0.8-3.89)	2.5(0.57-11.02)
Tigre	3	6	2.5(0.57-10.77)	1.96(0.07-23.32)
Agnuak	19	94	1.0	1.0
**Number of life births**				
Six and above	32	92	1.16(0.62-2.18)	1.68(0.56-5.02)
Four up to five	3	3	3.33(0.63-17.8)	8.34(0.43-160.81)
Two up to three	10	15	2.22(0.87-5.67)	*12.6(2.24-71.01)
One	17	69	0.82(0.4-1.69)	0.71(0.23-2.20)
Zero	21	70	1.0	1.0
**MTCT of HIV occurs during breast feeding**				
No	47	89	^*^2.33(1.41-3.87)	1.48(0.53-4.18)
Yes	36	159	1.0	1.0
**Prevention of mother to child transmission of HIV as perceived benefit of HIV testing during ANC**				
No	46	99	^*^1.88(1.14-3.11)	0.95(0.32-2.88)
Yes	37	150	1.0	1.0
**Discussed with husband and sought agreement for HIV testing at home prior to the current ANC visit**				
No	69	128	*4.66(2.5-8.7)	*8.7(3.06-24.70)
Yes	14	121	1.0	1.0
**Respondents impression on pre-test counseling**				
Fair	18	15	*6.2(2.58-14.7)	2.8(0.77-10.13)
Good	47	151	1.6(0.85-2.99)	1.1(0.42-3.09)
Very good	16	82	1.0	1.0
**Divorce with husband as perceived response following HIV positive result of pregnant woman**				
Yes	29	40	*2.81(1.5-4.93)	10.7(3.57-31.83)
No	54	209	1.0	1.0
**Disclosure of HIV test result to husband by the counselor as preferred way of hearing HIV test result**				
Yes	19	27	*2.7(1.40 - 5.31)	*6.9(2.21-21.24)
No	48	186	1.0	1.0

* represents p - value < 0.05.

**Table 4:** Knowledge factors associated with refusal of HIV testing during ANC, Gambella Region, 2008

**Variables**	**Not-HIV tested**	**HIV tested**	**COR (95% CI)**	**P-Value**
**HIV transmits from one person to another**				
I don’t know	3	9	1.04(0.27-3.92)	0.96
No	4	3	4.14(0.91-18.9)	0.07
Yes	76	236	1.0	
**Knowledge status on ways of HIV transmission**				
Not Knowledgeable	15	44	1.03(0.54-1.96)	0.93
Knowledgeable	68	205	1.0	
**Knowledge status on means of HIV prevention**				
Not Knowledgeable	35	100	1.09(0.66-1.8)	0.75
Knowledgeable	48	149	1.0	
**When MTCT could occur**				
During pregnancy				
No	46	109	1.6(0.97-2.63)	0.07
Yes	37	140	1.0	
During delivery				
No	54	134	1.57(0.94-2.63)	0.09
Yes	29	118	1.0	
During breast feeding				
No	47	89	*2.33(1.41-3.87)	*0.001
Yes	36	159	1.0	
**Means of Knowing one’s HIV status**				
HIV test of the blood				
No	7	14	1.55(0.6 - 3.97)	0.37
Yes	76	235	1.0	
**Benefits of HIV testing during pregnancy**				
**to know HIV status and seek medical attention**				
No	52	131	1.51(0.91-2.52)	0.11
Yes	31	118	1.0	
**To know HIV status and take preventive measures**				
No	43	114	1.27(0.77-2.09)	0.34
Yes	40	135	1.0	
**To prevent mother to child transmission of HIV**				
No	46	99	*1.88(1.14-3.11)	*0.01
Yes	37	150	1.0	

* represents significant association.

### Factors associated with HIV testing among ANC attendees: attitude on PMTCT

Attitude factors were found to be associated with refusal of HIV testing. The odds of refusing HIV testing among the respondents who agree with the statement “a pregnant mother shall not undergo HIV testing at the time of her ANC visit unless she has got her husband/partner agreement for HIV test” is 2.75 times more than those who disagree (COR = 2.75, 95% CI = 1.53, 4.96) (Table 5). Likewise, the odds of refusal of HIV testing among those who agree with the statement “I consider HIV/AIDS as a threat causing fear up on me” was almost two times more than those who disagree (COR = 2.13, 95% CI = 1.09- 4.18) (Table 5).

**Table 5:** Attitude related factors associated with refusal of HIV testing during ANC, Gambella Region, 2008

**Variables**	**Not-HIV tested**	**HIV tested**	**COR (95% CI)**	**P-Value**
**“HIV testing is needed only for those pregnant women who are/have been engaged in sexual contact other than their husband/partner ”**				
Agree	2	24	0.24(0.06-1.03)	0.06
Neither agree nor disagree	5	6	2.38(0.71-8.02)	0.16
Disagree	75	214	1.0	
**“A pregnant woman shall not undergo HIV testing at the time of her ANC visit unless she has got her husband/partner agreement of the test”**				
Agree	26	39	*2.75(1.5-4.96)	*0.001
Neither agree nor disagree	9	13	*2.86(1.2-7.08)	*0.02
Disagree	47	194	1.0	
**“People who are living with or thought to live with HIV must be separated and lead their own life in the absence of social relations with their community to prevent the transmission of HIV.”**				
Agree	13	48	0.76(0.39-1.5)	0.43
Neither agree nor disagree	2	10	0.56(0.12-2.64)	0.47
Disagree	67	189	1.0	
**“ I feel that I am at increased risk of HIV infection”**				
Disagree	26	109	0.58(0.22-1.54)	0.27
Neither agree nor disagree	42	120	0.85(0.33-2.19)	0.74
Agree	7	17	1.0	
**“I consider HIV as a threat causing fear upon me”**				
Agree	21	38	*2.13(1.1-4.18)	*0.027
Neither agree nor disagree	29	97	1.16(0.65-2.07)	0.63
Disagree	29	112	1.0	

* represents significant association.

### Factors associated with HIV testing among ANC attendees: cultural practices

Table 6 shows main cultural factors associated with refusal of HIV testing,The odds of refusing HIV testing among those who did not discuss with their husband about HIV testing was about eight and half times more than those who had discussed with their husband after controlling the effect of other variables (COR = 4.66, 95% CI = 2.49-8.71; AOR = 8.7, 95% CI = 3.06, 24.70) (Table 3). Of those who responded that they did not discuss with their husband about HIV testing, the odds of refusing HIV test among those who claim cultural barrier as a reason for no discussion with their husband about HIV was about three and half times more than those who did not claim cultural barrier as a reason for no discussion (COR = 3.69, 95% CI = 1.19,11.49). In the same analysis, the odds of refusing HIV test among those who reported that they did not discuss with their husband to avoide suspicion up on themselves, is three times more than those who did not claim this as a reason(COR = 3.07, 95% CI = 1.24-7.61). Those who claimed fear of their husbands’ reaction in terms of anger and attack following HIV positive result as a reason for no discussion were three times more likely to refuse HIV testing than their counterparts (COR = 3.0, 95% CI = 1.26, 7.19).

**Table 6:** Practices associated with refusal of HIV testing during ANC, Gambella Region, 2008

**Variables**	**Not-HIV tested**	**HIV tested**	**COR (95% CI)**	**P-Value**
**Number of ANC visits on the current pregnancy**				
One	58	177	0.84(0.34-2.1)	0.72
Two up to three	18	54	0.86(0.31-2.4)	0.77
Three up to four	7	18	1.0	
**Discussion with husband prior to HIV test during ANC**				
No	69	128	*4.66(2.5-8.7)	*0.001
Yes	14	121	1.0	
**Identified reasons for not discussing about HIV test at home with husband prior to the current ANC visit**				
**Our culture does not allow to discuss this kind of issues with my husband/partner**				
**Yes**	9	5	*3.69(1.2-11.5)	*0.02
**No**	60	123	1.0	
**To avoid any possible suspicion that arises from my husband/partner up on me**				
**Yes**	13	9	*3.07(1.2-7.6)	*0.02
**No**	56	119	1.0	
**Fear in case the results turns out to be HIV positive**				
**Yes**	4	5	1.51(0.4-5.8)	0.55
**No**	65	123	1.0	
**Fear of my husbands’ reaction in terms of anger and attack following HIV positive test result**				
**Yes**	14	10	*3.0(1.26-7.19)	*0.01
**No**	55	118	1.0	
**Husband’s absence at home at time of the current ANC visit**				
**Yes**	17	38	0.77(0.4-1.5)	0.45
**No**	52	90	1.0	
**Lack of knowledge about the presence of HIV counseling and testing service for pregnant mothers in local H/Institutions**				
**Yes**	13	50	*0.36(0.18-0.7)	*0.004
**No**	56	78	1.0	
**Possibility to come with husband for HIV test in the future**				
I don’t know	26	25	*4.5(2.25-8.36)	*0.00
No	9	16	*2.4(1.02-5.85)	*0.05
Yes	48	208	1.0	
**Respondents impression on pre-test counseling for HIV**				
Fair	18	15	*6.2(2.58-14.7)	*0.00
Good	47	151	1.6(0.85-2.99)	0.15
Very good	16	82	1.0	
**Privacy is maintained in the counseling room**				
I am not sure	28	23	*5.2(2.70-9.79)	*0.00
No	3	3	4.3(0.84-21.9)	0.08
Yes	52	223	1.0	
**Husband’s response towards HIV positive result of pregnant mothers**				
**Provision of support in one way or the other**				
No	64	152	*2.15(1.21-3.8)	*0.01
Yes	19	97	1.0	
**Insulting saying,’It is you who have brought disease”**				
Yes	23	51	1.49(0.84-2.6)	0.17
No	60	198	1.0	
**Attack and frequent beatings**				
Yes	23	47	1.6(0.93-2.93)	0.09
No	60	202	1.0	
**Divorce**				
Yes	29	40	*2.81(1.5-4.93)	*0.00
No	54	209	1.0	
**Would stop financial assistance**				
Yes	6	5	*3.8(1.13-12.8)	*0.03
No	77	244	1.0	
**Suggested sex of the counselor**				
I don’t mind for both sexes	49	161	0.65(0.33-1.3)	0.21
Male	2	18	0.2(0.05-1.15)	*0.07
Female	16	34	1.0	
**Suggested way of hearing HIV test result**				
Face -to - face communication with the counselor				
No	11	7	*5.7(2.14-15.6) *0.001	
Yes	56	206	1.0	
**Disclosure of HIV test result for husband by the counselor as preferred way of hearing HIV test result**				
Yes	19	27	*2.7(1.4 - 5.31) *0.003	
No	48	186	1.0	

* represents significant association.

Those respondents, who would not expect to get their husband support following their HIV positive result, were almost two times more likely to refuse HIV testing than those who expect to receive such treatment by their husband (COR = 2.15, 95% CI = 1.21, 3.80) (Table 6).

On the other hand, those who expect divorce as possible reaction of their husband following HIV positive result were almost ten and half times more likely to refuse HIV testing than those who would not expect divorce from their husband after controlling possible confounders (COR = 2.81, 95% CI = 1.50,4.93; AOR =10.7, 95% CI = 3.57, 31.83) (Tables 6 and 5).

### Factors associated with HIV testing among ANC attendees: service provision

In order to identify service provision related factors influencing HIV testing among ANC attendees, respondents were probed with questions focusing on their satisfaction level on the contents of pre-test counseling service and issue of privacy at the time of counseling. Those respondents who reported their impression on the contents of the pre-test counseling service being fair were six times more likely to refuse HIV testing than those who stated their impression on the pre-test counseling service being very good (COR = 6.2, 95% CI = 2.58, 14.7). Moreover, 80.7% of accepters and 85.5% of refusals suggested the need of improvement of pre-test counseling service being given to ANC attendees. In the same context, respondents who were not sure about the maintenance of privacy at the time of counseling were five times more likely to refuse HIV testing as compared to those who responded that privacy was maintained at the time of counseling (COR = 5.2, 95% CI = 2.70, 9.79) (Table 6).

### Main findings of the FGD

All discussants of the FGD agreed that HIV testing during pregnancy is beneficial to prevent mother to child transmission of HIV/AIDS. The other benefit mentioned by the discussants was the opportunity that testing provides pregnant women to know their own HIV status to start taking drugs that will make them live longer. The participants mentioned various barriers for HIV testing service utilization. The majority of the discussants had agreed on fear associated with the possibility of becoming HIV positive as a reason for refusal of HIV testing. During the discussion, it was also learnt that pregnant woman worry about the stress that would be induced when they think of coping mechanisms to address all the difficulties of living while being HIV positive. A difficult task, according to many of the discussants, not dared especially at the time of pregnancy. Of particular importance is the stigma and discrimination attached with HIV positive result. One discussant disclosed the degree of stigma in the study area as follows, *“…For instance, in our neighborhoods let alone eating and drinking together with people living with the virus, access to house rent was being denied even at times when they can afford to pay…”.*

The participants agreed on lack of punctuality by health professionals as a main barrier encountered at health facilites. One participant expressed her feeling in the following way, “…*many pregnant women hate and complain of sitting for long time to get the services and prefer not to come rather than suffering a lot with tiredness and discomfort*”.

Majority of the discussants agreed that they fear to discuss the idea of HIV testing with their husband. This is because of their awareness that their husbands are not interested for HIV testing. As well, they feared incrimination attached to ANC attendees as the one responsible for the bring in of the virus. A discussant with 20 years old, secondary school level, had HIV tested, stated that, “…*if an ANC attendee test by her own decision and inform herr husband to be tested, too, the first thing she would face is confrontation from her husband who needs explanation as to why she was tested without informing him…. After that, if the wife is found to be HIV positive, then she would be blamed as the one with sexual promiscuity and deviant behavior to import the virus*.”

One participant with the age of 35 years, had five children, and not tested, stated that, “… *majority of the husbands refuse to be tested together and if they do they would be tested secretly and start to take life longing medication with out informing their wives. This is because, if they were known to be HIV positive while their wives are negative, then the families of their wives would put a demand up on him for compensation claiming that their daughter will die from the illness that she acquired from him*”.

## Discussion

In this study, pregnant women who had 2–3 live births were more likely to refuse HIV testing as compared to those women who had no live births in the past. This might be explained by that such pregnant women have spent a longer period in their relationship with their husband and thus may feel secured from HIV infection and therefore may see no reason for HIV testing. Similar finding was obtained in a study conducted in Mbale district, Uganda and other studies. In the Uganda study, HIV testing was significantly lower in pregnant women with two and more live births in the past [13-15].

Knowledge about the transmission of HIV/AIDS from one person to another was found to be almost the same between HIV tested and not tested pregnant women on their recent ANC visit. This finding is consistent with the study done in Harar town about the intention of breastfeeding practice in the context of HIV/AIDS and with the study conducted in Jimma on assessment of KAP in lactating mothers about VCT and feeding of infants born to HIV positive women [16,17]. This finding was also strengthened by qualitative finding in which majority of the discussants, including those tested and not tested, agreed on main ways of HIV transmission except that only few agreed on MTCT.

Perceived partner response as divorce towards HIV positive result was found to be positively associated with refusal of HIV testing in this study. This is consistent with a study conducted in Vietnam and other countries which indicated that pregnant women who worry about their husband reaction in case he learnt that they were tested positive were refusing HIV testing during their ANC visit [18-20]. Moreover, our study finding is consistent with a study conducted in Uganda, Tanzania, Zambia and Vietnam. The study indicated that pregnant women who worry about their husband reaction in case he learnt that they were tested positive were refusing HIV testing during their ANC visit [18,21-23]. In a multicoated study in Kenya, Tanzania and Trinidad, it was indicated that participants in sero discordant female-positive couples were most likely to report the break-up of sexual relationship [24-26]. Such finding that reveal perceived negative husband response, expressed as divorce in our context, being an independent predictor for refusal of HIV testing could be explained with the possibility that the study participants are living in a kind of society where HIV testing of pregnant women at antenatal clinics has perceived social implications. Hence, informing their husband/partner about their own HIV positive status can be a major undertaking for most of them that perceived negative husband response for HIV positive ANC attendees become an important determinant for refusal of HIV testing in ANC set up. The qualitative finding had also shown that divorce would be the response of husband following HIV positive result, which pregnant women feared a lot. Such finding in our study could be explained with the possibility that the study participants are living in a kind of society where HIV testing at ANC has perceived social implications, that is, divorce. One participant in the qualitative study had stated that, *“If HIV virus is found in my blood, my husband would divorce with me. Let alone divorce…even there might be a possibility that he would quarrel with my families saying that I am the one who had infected him.”*

It was shown that lack of discussion with husband to seek his approval before HIV testing being an independent predictor for refusal of HIV testing. This is also consistent with a study conducted in Mbale district of Uganda, Kassena-Nankana district of Ghana and Zimbabwe which indicated that women with husbands being their primary confidant were independently associated with HIV testing than their counterparts [13,27,28]. Other study that is conducted in rural and urban Uganda also indicated that the strongest predictor for lack of willingness to accept an HIV test was the woman’s perception that her husband would not approve of her testing for HIV. Women who thought their husbands would approve were almost six times more likely to report a willingness to be tested compared to those who thought their husbands would not approve [19,29]. Such finding could be explained with the fact that women need to discuss with their husband about testing before accessing the service for fear of the blaming of not asking permission and other social consequences that they would face from their spouses following HIV positive test result. The fear is high with the fact that women get more economic and emotional support from their husband that is anticipated to cease following their HIV positive result [30]. Therefore, not to lose the benefits, they would put consultation of their husband at the forefront before deciding to test. Besides, the qualitative study had indicated that those who did not discuss with their husband tend to refuse HIV test.

In this study, disclosure of HIV test result to husband by the counselor was found to be positively associated with refusal of HIV testing. The finding in our study could be explained with the possibility that the study participants would find it difficult to share their test result with their husband because of their anticipation of blame and rejection. Besides, as it has been seen on the qualitative finding, cultural factors like the role of polygamy marriage in creating rival wives who want to keep their test result as secret from their husband for fear of losing him and the demand for compensation payment from the one assumed to bring the disease may enforce no test result disclosure. There fore, suggestion of test disclosure to husband may result in refusal for HIV testing. One discussant in the FGDs mentioned that, “*….for instance, if you take me I am the third wife for my husband. If one among us gets HIV tested, no one of us would tell HIV positive test result to our husband* …”

The study also showed that large proportion of the respondents from both groups indicated the need of improvement of HIV counseling service to ANC attendees. The figures show that the quality of counseling service was jeopardized in one way or the other, which literally speaking, brought the felt need for its improvement by both study groups. In addition, the qualitative finding indicated that lack of confidentiality for test result, lack of punctuality for health professionals as prevailing service provision related factors for refusal of HIV testing.

The limitation of this study is the possibility of selection bias that might have been introduced when ANC attendees refuse to participate. But the number of refusals to participate was to small and the bias may not have any effect on the study. Another limitation of the study is that there might be injection of interview administered bias, since the interviewers were health professionals. To minimize such bias intensive training has been given to the data collectors and supervisors. Not withstanding these limitations, we believe that our study has very important findings for strengthening of PMTCT implementation in Gambella region and areas with similar set up.

In conclustion, the following factors were identified as independent determinants for refusal of HIV testing: Reproductive characteristic (parity 2–3); perceived stigma from husband in the form of divorce; gender difference in the decision for service utilization; feared consequences of test disclosure; and cultural factors prevailing in a given ethnic group.

### Recommendations

The following recommendations are forwarded based on the study findings:

· Provision of innovative information and education on the pre-test session to address barriers of testing for those pregnant woman having two or more children during their ANC visit; women empowerment through decreasing the gap in education, providing information and skills, and improving women’s access to economic resources and assets so that to enable them to control over their own decision on accessing service utilization; couple counseling facilitated through couple-friendly ANC services could be taken as a strategy to minimize the difficulty that pregnant women face to disclose their HIV test result to their husband; fighting harmful traditional practices that are related with refusal of HIV testing in a given ethnic groups through community dialogue; and further qualitative study assessing the implication of socio-cultural factors in the context of HIV testing among ANC attendees is highly recommended.

## Competing interest

The authors declare that they have no competing interests.

## Authors’ contribution

WF and AW: participated in all steps of the study from its inception to write up. Both have reviewed and approved the submission of the manuscript.

## Author’s information

WF: is a public health expert graduated with distinction from Addis Ababa University. Currently, he is working as a program coordinator at UNICEF.

AW: is Associate Professor at the Department of Epidemiology and Biostatistics, School of Public Health of Addis Ababa University, Ethiopia. He has been teaching Biostatistics and Research methods for many years. He has worked as co-investigator and technical coordinator, for many trial studies and other researches. He published more than forty five peer-reviewed articles in international and national journals.

## References

[B1] UNAIDS, WHO AIDS epidemic update 2007 WHO, Geneva

[B2] MOH, HAPCO Single point HIV prevalence estimate 2007 MOH, Addis Ababa

[B3] Country Progress Report on HIV/AIDS Response 2012 Federal Democratic Republic of Ethiopia, Addis Ababa, Ethiopia

[B4] Ethiopian Demographic and Health Survey Addis Ababa Ethiopia and Calverton 2011 Centeral Statistical Agence and ICF International, Maryland USA

[B5] Kumbi S Reasons for refusal of HIV testing in two Ethiopian antenatal clinics 4970 International AIDS Soceity; Poster Exhibition: The XIV International AIDS Conference: Abstract no, 4970

[B6] Demissie A Deribew A Abera M Determinants of acceptance of voluntary HIV testing among antenatal clinic attendees at Dil Chora Hospital, Dire Dawa, East Ethiopia Ethiop J Hlth Dev 2009 23 2 141 147

[B7] GRHB Gambella Regional Health Bureau annual report 1999 , Gambella

[B8] WHO Anti retro viral drugs for treating pregnant women and preventing HIV Infection in infants in resource limited settings 2006 Recommendation for Public health approach,

[B9] MOH, HAPCO Guidelines for Prevention of Mother to Child Transmission Of HIV in Ethiopia 2007, Addis Ababa

[B10] Ethiopia Demographic and Health Survey Addis Ababa, Ethiopia and Calverton 2006 Central Statistical Agency and ORC Macro, Maryland, USA

[B11] Abebe A Mitikie G Perception of High School Students towards Voluntary HIV Counseling and Testing, using Health Belief Model in Butajira, SNNPR Ethiop J Hlth Dev 2009 23 2 148 153

[B12] Getachew W Enqusilassie F Factors determining acceptance of VCT among pregnant women attending ANC in Addis Ababa Ethiopian Med J 2007 45 1 1 8 17642152

[B13] Charles AS Karamagi Antenatal HIV testing in rural eastern Uganda in 2003, Incomplete roll out of prevention of mother to child transmission of HIV program BMC Int Health Hum Rights 2006 6 6 10.1186/1472-698X-6-6 16670031 PMC1533856

[B14] Getachew W Fikre E Factors determining acceptance of VCT among pregnant women attending ANC in Addis Ababa Ethiopian Med J 2007 45 1 1 8 17642152

[B15] Haddis M Jerene D Awareness of antenatal care clients on Mother to Child Transmission of HIV infection and its prevention in Arbamich Hospital Ethiop J Heal Dev 2006 20 1 55 57

[B16] Assegd M Assessment of intention and practice of VCT and Infant Feeding in the context of HIV/AIDS among lactating mothers in Harar Town Ethiopia 2006 AAU MPH Thesis March,

[B17] Hailu C Assessment of KAP among mothers about VCT and feeding of Infants born to HIV positive women in Jimma town Ethiopia 2005 AAU MPH Thesis,

[B18] Dinh TH Kamb ML Factors associated with reluctance to accept HIV Testing in pregnant women in Vietnam Int Conf AIDS 2004 15 Abstract No ThPeC7304 11 16

[B19] Francis B Michael M Barriers to the implementation of programs for the prevention of mother-to-child transmission of HIV: A cross-sectional survey in Urban and Rural AIDS Res Ther 2005 2 10 10.1186/1742-6405-2-10 16255776 PMC1277814

[B20] Rosa H Barriers for HIV testing during the pregnancy Rev Saúde Pública 2006 40 2 220 225 10.1590/s0034-89102006000200006 16583031

[B21] Bakari JP McKenna S Myrick A Rapid Voluntary testing and counseling for HIV, Acceptability and feasibility in Zambian Antenatal care clinics, Lusaka, Zambia Ann N Y Acad Sci 2000 918 64 76 11131736 10.1111/j.1749-6632.2000.tb05475.x

[B22] Pool R Nyanzi S Whitworth JA Attitude of pregnant women towards VCT in rural south-west Uganda AIDS Care 2001 13 605 615 10.1080/09540120120063232 11571007

[B23] Westheimer EF Urassa W Msamanga G Acceptance of HIV testing among pregnant women in Dar-es-salaam,Tanzania J Acquir Immune Defic SyndrSept 2004 37 1 1197 1205 10.1097/01.qai.0000120806.43677.ff 15319681

[B24] Covadia HM Access to Voluntary Counseling and Testing for HIV in Developing Countries Ann N Y Acad Sci 2000 918 57 63 11131735 10.1111/j.1749-6632.2000.tb05474.x

[B25] De Paoli MM Manongi R Kleep KI Factors influencing acceptability of voluntary counseling and HIV-testing among pregnant omen in Northern Tanzania AIDS CARE May 2004 16 4 411 425 10.1080/09540120410001683358 15203410

[B26] Emily Westheimer F Willy U Gernard M Acceptance of HIV testing among pregnant women in Dar-es-salaam,Tanzania J Acquir Immune Defic SyndrSept 2004 37 1 1197 1205 10.1097/01.qai.0000120806.43677.ff 15319681

[B27] Baiden F Remes P Voluntary counseling and HIV testing for pregnant women in the Kassena-Nankana district of northern Ghana: Is couple counseling the way forward? AIDS CARE Jul 2005 17 5 648 657 16036251 10.1080/09540120412331319688

[B28] Perez F Mukotekwa T Miller A Implementing a Rural program on prevention of mother-to-child transmission of HIV in Zimbabwe. The first 18 months of experience Trop Med Int Health 2004 9 7 774 783 10.1111/j.1365-3156.2004.01264.x 15228487

[B29] Kumar A Rocheste E al GMe. Antenatal Voluntary Counselling and Testing for HIV in Barbados; success and Barriers to implementation Rev Panam Salud Publica 2004 15 4 242 248 10.1590/S1020-49892004000400004 15193179

[B30] Knut F Seter S A randomized trial on acceptability of Voluntary counseling and testing Trop Med Int Health May 2004 9 5 566 572 10.1111/j.1365-3156.2004.01231.x 15117300

